# Novel In Vivo CometChip Reveals NDMA-Induced DNA Damage and Repair in Multiple Mouse Tissues

**DOI:** 10.3390/ijms231911776

**Published:** 2022-10-04

**Authors:** Norah A. Owiti, Joshua J. Corrigan, Lee J. Pribyl, Jennifer E. Kay, Bevin P. Engelward

**Affiliations:** 1 Department of Biological Engineering, Massachusetts Institute of Technology, Cambridge, MA 02139, USA; 2 Silent Spring Institute, Newton, MA 02460, USA

**Keywords:** in vivo CometChip, *N*-nitrosodimethylamine, DNA repair, mice

## Abstract

The comet assay is a versatile assay for detecting DNA damage in eukaryotic cells. The assay can measure the levels of various types of damage, including DNA strand breaks, abasic sites and alkali-sensitive sites. Furthermore, the assay can also be modified to include purified DNA glycosylases so that alkylated and oxidized bases can be detected. The CometChip is a higher throughput version of the traditional comet assay and has been used to study cultured cells. Here, we have tested its utility for studies of DNA damage present in vivo. We show that the CometChip is effective in detecting DNA damage in multiple tissues of mice exposed to the direct-acting methylating agent methylmethane sulfonate (MMS) and to the metabolically activated methylating agent *N*-nitrosodimethylamine (NDMA), which has been found to contaminate food, water, and drugs. Specifically, results from MMS-exposed mice demonstrate that DNA damage can be detected in cells from liver, lung, kidney, pancreas, brain and spleen. Results with NDMA show that DNA damage is detectable in metabolically competent tissues (liver, lung, and kidney), and that DNA repair in vivo can be monitored over time. Additionally, it was found that DNA damage persists for many days after exposure. Furthermore, glycosylases were successfully incorporated into the assay to reveal the presence of damaged bases. Overall, this work demonstrates the efficacy of the in vivo CometChip and reveals new insights into the formation and repair of DNA damage caused by MMS and NDMA.

## 1. Introduction

The mammalian genome is constantly subjected to DNA damaging agents and it is critical to repair damaged DNA because unrepaired lesions can be mutagenic, thus promoting cancer [1,2]. Alkylating agents are one of the broadest classes of DNA damaging agents, creating adducts that contain one or more carbons. Methyl methanesulfonate (MMS) and *N*-nitrosodimethylamine (NDMA) create methylated bases, including 7-methylguanine (7MeG), 3-methyladenine (3MeA), and *O*^6^-methylguanine (*O*^6^MeG), for which the latter two lesions have been shown to be mutagenic, toxic, and carcinogenic [3,4,5,6,7]. Unlike MMS, NDMA is an indirect-acting methylating agent that requires metabolic activation by cytochrome P450-2E1 (CYP2E1) to form a DNA-reactive breakdown product, namely the methyldiazonium ion [8,9,10]. NDMA is a potent carcinogen in animal models, causing liver, lung, and kidney tumors [11], and NDMA has been found in contaminated medications and municipal water supplies [12,13,14,15,16]. More recently, NDMA was linked to a childhood cancer cluster in Wilmington, MA, and the source of the NDMA was traced to the Olin Chemical Superfund Site [17,18]. Given the public health relevance of NDMA, animal models are currently being used to deepen our understanding of the mechanisms of action of NDMA, which in turn will enable better prediction and prevention of NDMA-induced cancer (e.g., [6]). As such, it is valuable to be able to quantify NDMA-induced DNA lesions in various tissues. Here, we have established and employed the in vivo CometChip platform to reveal the presence of MMS- and NDMA-induced DNA damage in multiple mouse tissues.

Over the past three decades, the single-cell gel electrophoresis assay, more commonly known as the comet assay, has been widely used as a standard method to measure DNA damage in eukaryotic cells [19,20,21,22,23]. The alkaline comet assay measures DNA strand breaks and alkali-sensitive lesions, such as apurinic/apyrimidinic (AP) sites, which are converted into strand breaks under high pH [19]. This assay works on the principle that undamaged DNA is highly supercoiled and resists migration in an agarose gel, whereas nicked DNA contains loops that can be pulled away from the nucleoid, giving the appearance of a comet [21,24]. To do the traditional assay, cells are embedded in agarose on glass slides, cells are lysed, the DNA is denatured at a high pH, and the resulting nucleoids are subjected to alkaline electrophoresis [19,20,21]. The comet assay is highly versatile and is widely used in genotoxicity testing (e.g., [24,25,26,27]) as a tool for assessing DNA damage in human cells (e.g., [28,29,30,31]) and in eco-genotoxicological studies (e.g., [32,33]). While the in vitro comet assay is used to analyze DNA damage in cultured cells, the in vivo comet assay can reveal DNA damage in tissues from animals. Following a six-year validation study that was finalized in 2012 [34], the in vivo comet assay was adopted by the Organization for Economic Co-operation and Development in 2016 (OECD test guideline no.489) [35].

While effective, the traditional comet assay is low throughput because a glass slide is necessary for each condition. The assay is also laborious, as a user must analyze at least 100 comets individually using comet analysis software. The CometChip is a high-throughput version of the comet assay that uses an agarose microwell array produced by pressing a mold with micron-scale pegs onto molten agarose. Once the molten agarose gelates, the stamp is removed to reveal an array of microwells that are ~30–50 μm in diameter and are spaced ~240 μm from each other. A bottomless 96-well plate is then clamped onto the agarose to create 96 macrowells, each containing ~300–400 microwells at its base. Cell suspensions are added to macrowells, and the cells drop into the microwells by gravity. Excess cells are removed by shear force, resulting in an array of micropatterned cells that can then be processed using standard comet assay conditions. There are several advantages of the CometChip assay over the traditional comet assay. First, the 96-well format allows for multiple samples to be analyzed at the same time on a single chip instead of a multitude of glass slides, thus reducing labor and sample-to-sample variation [36,37]. Second, the approach reduces bias because the microwell array suppresses overlapping comets, and so the user does not need to identify appropriate comets to analyze. Third, because cells are in the same focal plane, multiple comets (>100) can be captured in a single image. Finally, dozens of comets can be automatically analyzed in a single image using in-house or commercial software, avoiding the need for analyzing thousands of comets one by one, which greatly reduces time and effort of analysis [36,37]. The CometChip was originally developed in the laboratories of Engelward and Bhatia, and was first described in a publication in 2010 [36]. Since then, the method has been demonstrated to be effective in more than three dozen publications. (Recent publications include: [38,39,40,41,42,43,44,45]). When cells are exposed to methylating agents, DNA strand breaks and alkali-labile sites can arise as transient intermediates during base excision repair (BER). For example, 3-methyladenine and 7-methylguanine are removed by the alkyladenine DNA glycosylase (AAG), also known as N-methylpurine DNA glycosylase (MPG), to initiate the BER pathway. Following removal of damaged bases, abasic sites are nicked by AP endonucleases prior to repair synthesis and ligation. As such, BER creates transient AP sites and single strand breaks that are detectable using the alkaline comet assay. Additionally, the comet assay has been modified to enable detection of a variety of DNA base lesions. Specifically, DNA in nucleoids is incubated with purified glycosylases, which convert base lesions (that do not affect DNA migration in agarose) into AP sites. Since AP sites are susceptible to cleavage under alkaline conditions, glycosylases convert undetectable base lesions into detectable strand breaks [27,46,47,48]. Relevant to this study, the formamidopyrimidine-DNA glycosylase (Fpg) can release oxidized purines and the human alkyladenine DNA glycosylase (hAAG) can remove 3-methyladenine and 7-methylguanine.

The traditional in vivo comet assay is well established and has been successfully used to measure DNA damage in many different rodent tissues (e.g., [49,50,51,52]). Furthermore, many studies included glycosylases, such as Fpg, to reveal the presence of modified DNA bases. The CometChip has been widely adopted by many labs; however, most of these studies have focused on cultured cells. Given the advantages of the CometChip, we set out to determine whether (i) cells from different organs could be loaded onto the CometChip, (ii) DNA damage from tissues can be measured using the CometChip, and (iii) DNA repair enzymes can be incorporated into the CometChip to reveal modified DNA bases in mouse tissues. We further sought to determine whether the time of freezing tissue samples affects background DNA damage levels. Here, we show that these three objectives are achievable by demonstrating that the CometChip can detect DNA damage in a multitude of tissues obtained from mice that were exposed to DNA methylating agents (specifically, liver, lung, kidney, pancreas, brain and spleen). Furthermore, application of the approach revealed that NDMA-induced DNA damage can be detected in metabolically competent tissues, that DNA repair can be monitored over time, and that DNA damage persists for many days after exposure to NDMA.

## 2. Results

### 2.1. In Vivo CometChip Can Detect MMS-Induced DNA Damage in Multiple Mouse Tissues

One of our main objectives was to test the efficacy of the CometChip for the in vivo comet assay, wherein DNA damage in the animal, rather than DNA damage introduced in vitro, can be assessed. We elected to start by exposing mice to MMS, a direct-acting methylating agent, so that we could explore the ability to detect DNA damage in a multitude of tissues. High concentrations of MMS are commonly used as a positive control in the in vivo comet assay (e.g., [49,50,53]). For these studies, we treated 15-day-old mice with 0, 5, 25, and 50 mg/kg MMS via intraperitoneal (IP) injection.

**Figure 1:**
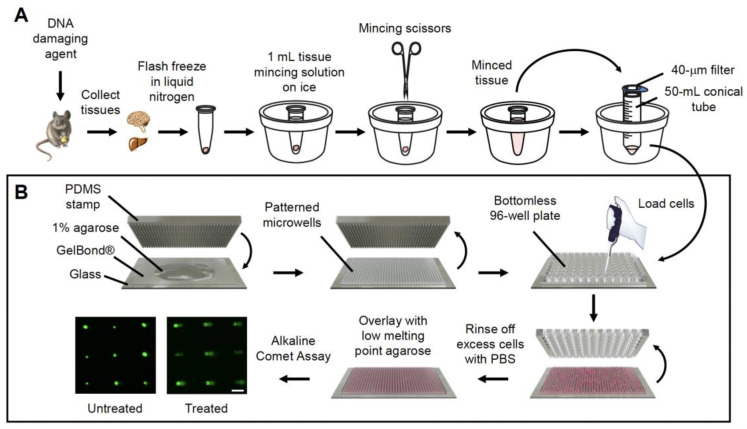
Schematic for the in vivo CometChip assay. (**A**) Mice are exposed to a DNA damaging agent, and tissues from various organs are collected and immediately flash frozen in liquid nitrogen. The tissues are transferred to −80 °C for long term storage until ready for the CometChip assay. To make cell suspensions, tissues are immersed in 0.5–1.0 mL of cold tissue mincing solution and gently minced on ice and cell suspensions are filtered through a 40-μm filter to remove debris. (**B**) To make the CometChip, 1% normal melting point agarose in PBS is prepared. A polydimethylsiloxane (PDMS) stamp with an array of micropegs is pressed into molten agarose solution on top of the hydrophilic side of a sheet of Gelbond film. The agarose is allowed to gelate and the PDMS stamp is removed to reveal an array of microwells. A bottomless 96-well plate is pressed on top of the agarose chip to form 96-macrowells. Filtered cell suspensions are loaded onto the CometChip and excess cells are rinsed with PBS. The loaded cells are capped with 1% low melting point agarose and the alkaline comet assay is performed as described in the Materials and Methods. Scale bar = 100 μm.

Unlike the traditional comet assay, the CometChip includes a cell loading step, wherein cells drop into microwells to create a cell microarray. We therefore set out to determine if cells from various tissues (namely, liver, lung, kidney, pancreas, brain, and spleen) can be loaded and analyzed effectively using the CometChip. Tissues were extracted from mice and immediately flash frozen in liquid nitrogen until ready for use. The tissues were subsequently minced on ice and filtered to attain single cells. Cells from each tissue were loaded onto the 96-well CometChip and incubated at 37 °C for 15 min to allow cells to settle into the microwells by gravity. After rinsing and overlaying with low melting point agarose, the CometChip was processed using standard conditions for the alkaline comet assay. Figure 1 shows the schematic overview for extracting cells from tissues and loading them onto the CometChip. 

**Figure 2:**
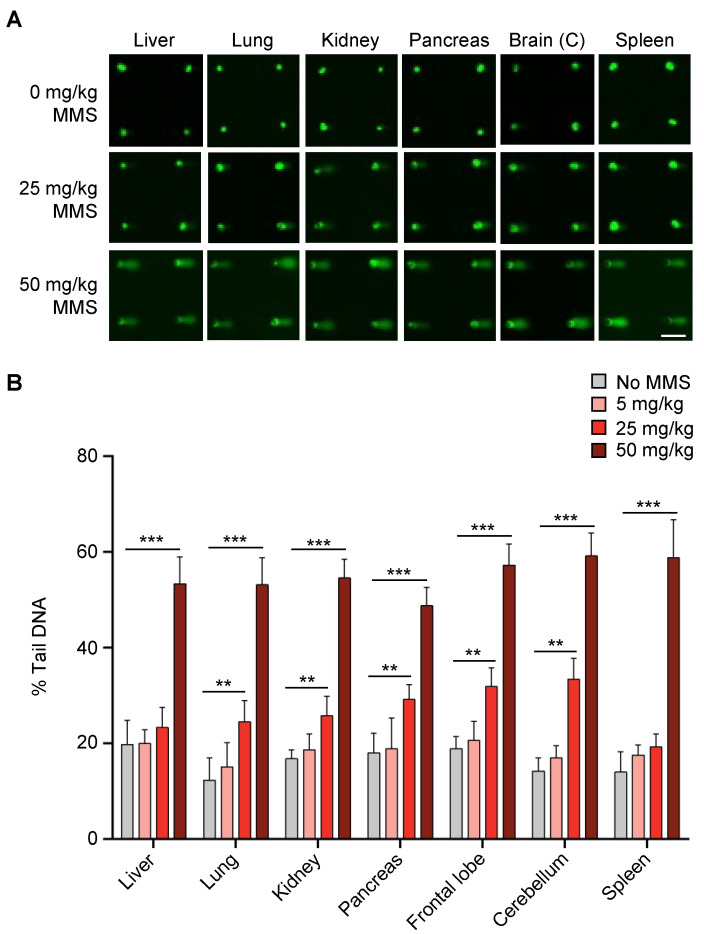
MMS-induced DNA damage can be detected in various mouse tissues using the CometChip. Mice were either sham treated or treated with MMS and various tissues were collected 3 h after injection and analyzed with the alkaline CometChip. (**A**) Example of fluorescent comet images from various mouse tissues exposed to varying doses of MMS. (**B**) Dose–response graphs of mice exposed to 0, 5, 25 or 50 mg/kg of MMS. All samples were analyzed in parallel on the same chip. Percent tail in DNA represented as mean of three mice ± SD. Unpaired Student’s *t*-test, ** *p* < 0.01, *** *p* < 0.001. Scale bar = 100 μm.

Cells from all the tissues were successfully loaded onto the CometChip (Figure 2A). It is worth noting that, due to the varying sizes of different cells, some tissues were more easily loaded onto the CometChip than others. For example, spleen cells are very small and had to be significantly diluted to reduce the number of cells because microwells with high numbers of cells per microwell had an increase in background damage (Figure S1A,B). In contrast, for the pancreas, we observed that many of the microwells were empty compared to other tissues, which is consistent with a lower concentration of cells due to the small size of the pancreas of young mice (Figure S1C). Nevertheless, we were able to get a sufficient number of microwells loaded to achieve at least 50 comets per condition for analysis.

One major advantage of the CometChip over the traditional comet assay is that its 96-well format reduces technical variability among samples [37], which is a common challenge for the traditional slide-based comet assay. Here, we were able to evaluate DNA damage levels in control mice and mice treated with three different doses of MMS all on the same chip. Visual inspection shows that comet tails are evident in most tissues from mice treated with 25 mg/kg and that all tissues gave rise to comets with long tails at 50 mg/kg (Figure 2A). Quantification of the results shows that there is no significant increase in DNA damage levels for mice treated with 5 mg/kg, whereas for 25 mg/kg MMS, almost all the tissues showed a statistically significant increase in percent tail DNA compared to controls, with the exceptions of liver and spleen (Figure 2B). In addition, positive results were observed for all tissues from animals treated with 50 mg/kg MMS (Figure 2B). These results show that the CometChip can be used to successfully analyze DNA damage present in a wide range of mouse organs, thus demonstrating that the in vivo CometChip is an effective approach for detecting DNA damage present in mouse tissues. 

### 2.2. Undetectable Base Damage Can Be Revealed Using DNA Glycosylases

The alkaline comet assay is effective for detecting single strand breaks, but it is not effective for detecting damaged bases because they do not affect DNA migration in agarose. To overcome this limitation, glycosylases can be incorporated into the comet assay after the lysis step and prior to electrophoresis to create alkali-labile sites at the sites of the bases they excise [27,46,48,54,55]. MMS predominantly creates 7-methylguanine and 3-methyladenine lesions [3,5,56] that are removed by the AAG glycosylase. Excision of damaged bases by AAG creates AP sites that get cleaved under alkaline conditions, leading to detectable strand breaks. The Fpg glycosylase from *E. coli* is best known for its ability to remove oxidized purines. Since alkylating agents can induce oxidative stress [57,58], we anticipated that Fpg would reveal the presence of damaged bases in cells from tissues of MMS-exposed mice.

**Figure 3:**
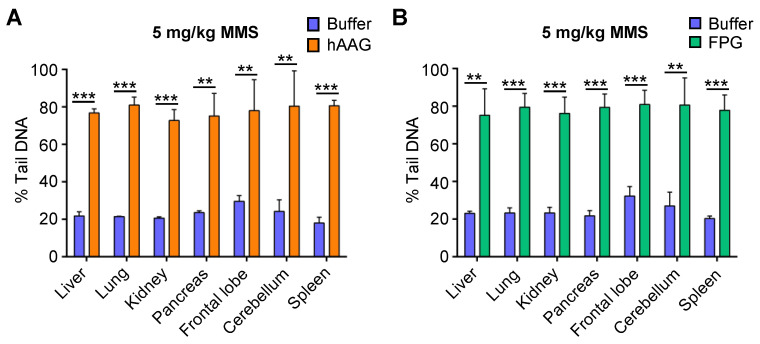
MMS-induced base lesions can be detected by the CometChip. Tissues from mice exposed to 5 mg/kg of MMS were analyzed with enzyme-modified alkaline CometChip. Results show DNA damage in various tissues using the CometChip modified by addition of (**A**) hAAG enzyme or hAAG buffer and (**B**) Fpg enzyme or Fpg buffer. Percent tail in DNA represented as mean of three mice ± SD. Unpaired Student’s *t*-test, ** *p* < 0.01, *** *p* < 0.001.

In initial studies using purified AAG, we analyzed MMS-treated mouse embryonic fibroblasts (MEFs) and human hepatocellular carcinoma, HepG2, cells in vitro. We observed a significant increase in percent tail in DNA (Figure S2A,B), which is consistent with previous studies that established the approach [47]. We therefore set out to investigate whether we could detect these MMS-induced modified bases using the in vivo CometChip. Although we did not detect DNA strand breaks in mice exposed to 5 mg/kg MMS (Figure 2B), we hypothesized that there may still be base damage present that had not yet been removed by a DNA glycosylase. Therefore, we asked if we would convert unrepaired DNA base lesions into detectable strand breaks at 5 mg/kg by adding hAAG or Fpg. Indeed, adding either hAAG or Fpg revealed a significant increase in DNA strand breaks for all tissues compared to buffer alone (Figure 3A,B). 

To confirm that the increase in strand breaks was due to the presence of unrepaired lesions induced by MMS, we also included tissue samples from sham-treated animals exposed to dimethyl sulfoxide (DMSO) only. We did not observe any significant increase in the number of strand breaks in the samples from mice that had only been exposed to DMSO (Figure S3A,B). These results show that the CometChip can be used to analyze the presence of modified DNA bases via the inclusion of DNA glycosylases.

### 2.3. Effect of Freezing Tissues on DNA Damage Levels 

The in vivo comet assay is generally performed using fresh tissues. However, processing many fresh samples from different organs at the same time is logistically challenging. Others have shown that it can be effective to freeze tissues prior to analysis [49,50,59], and thus we assessed the possible effects of freezing the tissues on DNA damage levels. To this end, we collected fresh liver tissues from animals that received 5 mg/kg of MMS and untreated controls. We flash froze a portion of the tissues in liquid nitrogen and then extracted cells from the fresh samples, immediately loaded them onto the CometChip, and performed the alkaline comet assay. The flash frozen tissues were stored at −80 °C for two weeks and then processed and analyzed as shown in Figure 1. For untreated controls, we observed a slight but statistically significant increase in percent tail DNA in the frozen liver tissues compared to the fresh livers (Figure S4). For the liver tissues from mice exposed to 5 mg/kg MMS, there was a slight but not statistically significant higher percent tail DNA in frozen tissues compared to fresh. Therefore, we conclude that while freezing tissues can potentially lead to an increase in percent tail DNA, the effect size is small, and so for all subsequent studies, we flash froze the tissues prior to analysis. 

### 2.4. The In Vivo CometChip Can Be Utilized to Detect DNA Damage Present in NDMA-Exposed Mice 

**Figure 4:**
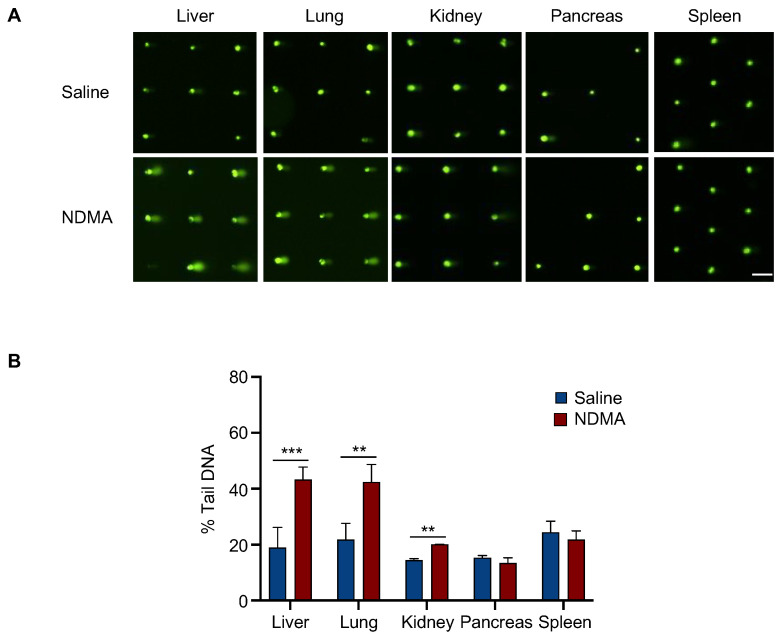
NDMA induces DNA damage in metabolically active mouse tissues 24 h after exposure. Mice were either sham treated or exposed to NDMA, and liver, lungs, kidneys, pancreas and spleen were collected 24 h after injection and analyzed with the alkaline CometChip. (**A**) Representative fluorescent comet images from various mouse tissues treated with NDMA. (**B**) Quantification of percent tail DNA showing the mean of three mice ± SD. Unpaired Student’s *t*-test, ** *p* < 0.01, *** *p* < 0.001. Scale bar = 100 μm.

Unlike MMS, NDMA requires metabolic activation by CYP2E1, which is expressed most highly in the liver. Previous studies in our laboratory showed that mice exposed to NDMA are susceptible to liver mutations and cancer [6]. Furthermore, there was an increase in micronuclei and γH2AX foci in hepatocytes, which is consistent with the creation of DNA double strand breaks. Here, we sought to utilize the in vivo CometChip to evaluate single strand breaks and alkali-sensitive sites induced by NDMA. Results show a significant increase in DNA damage in liver cells from mice 24 h after exposure to NDMA (Figure 4). It is known that CYP2E1, while being highest in the liver, is also expressed in the lungs and kidneys [60,61]. Only very low levels of CYP2E1 have been observed in other organs. To further explore NDMA-induced DNA damage in tissues other than liver, we analyzed DNA damage in the lungs, kidneys, pancreas and spleen. Consistent with the requirement for metabolic activation, we observed a significant increase in DNA strand breaks in the lung and kidney, but not in the spleen or pancreas where expression of CYP2E1 is low (Figure 4).

### 2.5. Evaluating Repair Using CometChip in Different Tissues Following NDMA Exposure

**Figure 5:**
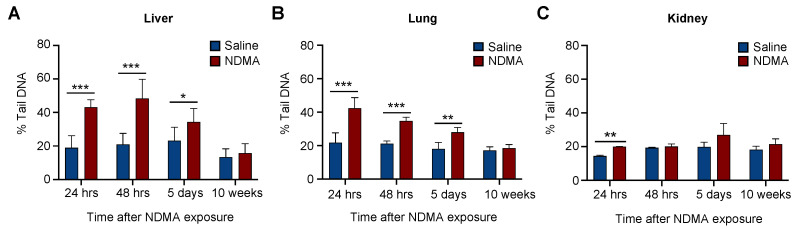
Repair of NDMA-induced strand breaks over time in the liver, lung and kidney. Mice were either sham treated or exposed to 10 mg/kg of NDMA, and liver, lungs and kidneys were collected 24 h, 48 h, 5 days and 10 weeks after injection and tissues analyzed with the alkaline CometChip. Bar graphs represents quantification of DNA strand breaks at all the time points in the (**A**) liver (**B**) lung and (**C**) kidney. Percent tail in DNA is represented as the mean of three mice ± SD. Unpaired Student’s *t*-test, * *p* < 0.01, ** *p* < 0.01, *** *p* < 0.001.

A valuable feature of the CometChip is that it enables investigation of DNA damage in dozens of samples at the same time allowing us to not only perform the in vivo CometChip analysis for multiple tissue types, but also for multiple time points in parallel. Mice were exposed to NDMA and liver, lungs and kidneys were collected at 24 h, 48 h, 5 days, and 10 weeks post-NDMA exposure (Figure 5). 

In the liver, percent tail DNA returned to baseline by 10 weeks (Figure 5A). Interestingly, the level of DNA damage at 48 h post-treatment was similar to that at 24 h, which is consistent with most of the DNA damage having not yet been fully repaired 48 h post-NDMA exposure and/or delayed metabolism of NDMA. We also observed significant DNA damage induced by NDMA 5 days post-NDMA exposure, but at a lower level compared to 24 h and 48 h, indicating repair of the NDMA-induced lesions. Regarding the lung tissue, we observed a reduction in DNA damage over time; however, we found that the DNA damage levels (percent tail DNA) were lower at 48 h compared to 24 h, which is consistent with active repair during that interval (Figure 5B). Similar to the results observed in the liver, the levels of DNA damage returned to baseline by 10 weeks in the lung. NDMA metabolism in the kidney is considerably lower than that of the liver or lung, so it was unsurprising that there was only a very small increase in DNA damage levels at 24 h. After 24 h, the levels of DNA damage were not statistically different from that of untreated mice (Figure 5C). Taken together, these results show that DNA repair in vivo can be assessed from frozen tissue samples using the CometChip, and that NDMA-induced DNA damage persists for many days post exposure.

### 2.6. Base Damage Present in Livers of NDMA-Exposed Mice Can Be Revealed Using hAAG and Fpg

**Figure 6:**
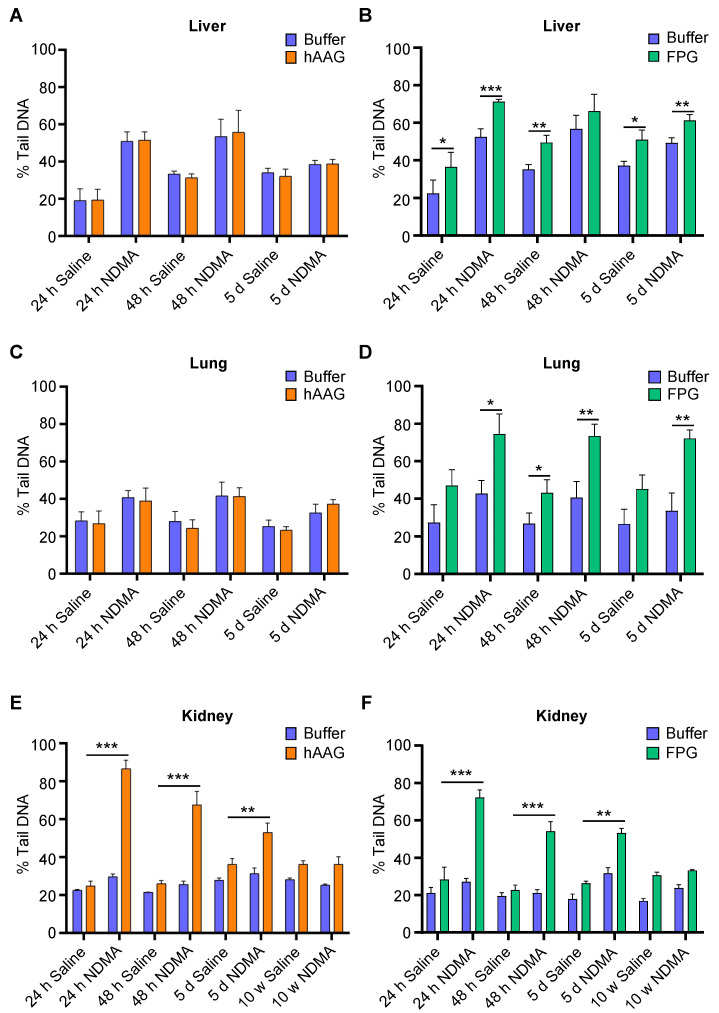
NDMA-induced base lesions can be detected by the CometChip. Mice were exposed to either saline or 10 mg/kg of NDMA, and liver, lungs and kidneys were collected 24 h, 48 h, 5 days and 10 weeks after injection. Cell suspensions from the tissues were analyzed with enzyme-modified alkaline CometChip. Results show DNA damage following assay modification with (**A**,**C**,**E**) hAAG enzyme or hAAG buffer and (**B**,**D**,**F**) Fpg enzyme or Fpg buffer. Percent tail DNA represented as mean of three mice ± SD. Unpaired Student’s *t*-test, * *p* < 0.01, ** *p* < 0.01, *** *p* < 0.001.

To evaluate base damage levels following NDMA exposure, we incorporated hAAG and Fpg enzymes. For liver and lung, we did not observe any increase in the percent tail DNA following incorporation of hAAG enzyme (Figure 6A,C), suggesting that the damaged bases are not present, which is consistent with the occurrence of repair. 

When Fpg enzyme was incorporated, there was a significant increase in percent tail DNA in both the liver and lung tissues that were exposed to NDMA compared to samples that were incubated in Fpg buffer only (Figure 6B,D). However, Fpg also led to an increase in percent tail DNA in tissues from saline-treated animals, which is consistent with normal levels of oxidative stress causing an increase in oxidized purines. In the kidneys, we observed a significant increase in percent tail DNA following the addition of hAAG and Fpg enzymes that decreased overtime, suggesting the presence of NDMA-induced base damage (Figure 6E,F). These results suggest that the removal of base lesions is slower in the kidney compared to their removal in the liver and lungs. Consistent with a lack of DNA strand breaks detected in the pancreas and spleen (Figure 4), we did not observe a significant increase in DNA damage in these tissues when we incorporated Fpg or hAAG (Figure S5A,B, which was expected given the low levels of CYP2E1 expression in these tissues. Overall, these data demonstrate that we can utilize the in vivo CometChip to evaluate DNA damage both as base lesions and BER intermediates including AP sites and strand breaks.

## 3. Discussion

DNA damage causes mutations that drive cancer, so it is important to have tools to detect DNA damage in tissues. Here, we have demonstrated that the CometChip can be effectively used to assess DNA damage in multiple mouse tissues using direct-acting and indirect-acting methylating agents, namely MMS and NDMA, respectively. NDMA is a potent carcinogen in animal models and is of serious concern, as millions of people are exposed to NDMA every day through ingestion of certain types of food, contaminated water supplies, and many have been exposed to NDMA in contaminated medications. It is therefore important to study the DNA damaging effects of NDMA. Using MMS, we demonstrated that the in vivo CometChip is effective for measuring DNA damage in liver, lung, kidney, brain, pancreas, and spleen tissues. Furthermore, we showed that the in vivo CometChip can be used to detect NDMA-induced DNA damage, and that the levels of DNA damage are consistent with the known tissue-specific variations in metabolic enzyme expression.

It is well established in multiple animal models that the carcinogenicity of NDMA is greatest in the liver, followed by the lungs, and then the kidneys [11,62]. These differences in carcinogenic potential can be partly explained by the variation of P450 expression in these organs [60,61], which directly correlates with cancer susceptibility. Consistent with these findings, following NDMA exposure, we observed DNA damage in the liver, lungs, and kidneys, but not in the pancreas or spleen, where P450s are expressed at very low levels. It has long been thought that the main cause of cancer following NDMA exposure is unrepaired *O*^6^-methylguanine, a highly mutagenic lesion that is removed via direct reversal rather than via BER. However, prior studies show that the mouse equivalent of hAAG plays a major role in modulating the effects of NDMA and other methylating agents in vivo, wherein mice lacking AAG are highly susceptible to mutations and cancer [6,7,63]. The work presented here complements those studies and points to the remarkable persistence of NDMA-induced DNA damage in the liver, with detectable lesions for as long as 5 days after exposure (Figure 5A,B). This result is consistent with either delayed activation of NDMA leading to formation of base damage more than a day after exposure and/or persistent BER intermediates. Further studies are needed to differentiate these two possibilities.

It is interesting that, in the liver, the addition of Fpg leads to a similar level of increase in percent tail DNA whether the animals were treated with saline or NDMA (Figure 6B). This observation is consistent with a model wherein oxidized bases are created by baseline levels of oxidative stress. Intriguingly, the outcome for kidney was quite different, with a high induction of Fpg-sensitive sites specifically for the NDMA-treated animals. One possibility is that NDMA is a more potent inducer of oxidative stress in the kidneys compared to the liver and lungs. An alternative explanation is that Fpg is taking out a different Fpg substrate, namely ring-opened 7-methylguanine (ro7MeG). However, ro7MeG is a minor lesion compared to 7MeG, so the strong induction of tail DNA is more likely the result of oxidized bases.

One of the key technical aspects to the CometChip is that cells need to be loaded into microwells that can hold one or a few cells each. Results show that all the tissues tested, including liver, lung, kidney, pancreas, brain and spleen, can be successfully loaded onto the CometChip, although the loading efficiency varies with the type of tissue. For example, cells from the spleen are very small and require extra dilution to avoid excessive loading of cells onto the chip for accurate quantification of the DNA damage (Figure S1). Using smaller microwells, such as the commercially available 30-μm instead of the 40-μm used here, may be an effective alternative to cell dilution. For smaller tissues, such as the juvenile mouse pancreas, fewer cells can be obtained. We observed that for the pancreas, there were many microwells without cells, even after mincing in a smaller volume of mincing solution compared to other tissues. It is possible that by mincing in an even smaller volume, a higher proportion of microwells could be loaded, but this carries the risk of not having enough for multiple replicates. Nevertheless, the procedure described in this manuscript for extracting and loading the cells onto the CometChip led to successful results with low background levels for all of the tissues analyzed. 

The CometChip enables analysis of multiple conditions at the same time on the same chip, significantly reducing assay variability and increasing efficiency [64]. In each individual figure presented here, all samples were run on the same chip, making it easier to study the effects of both chemical dose level (Figure 2) and repair efficiency (Figure 5) over time. For example, in Figure 2, we successfully ran all seven tissues with four different treatment conditions on the same chip. Each sample was tested in three macrowells, averaging approximately 450 comets per sample analyzed in each experiment. As such, in a single experiment that took only a few days, we analyzed >10,000 comets. The enormous amount of data that can be collected for each of these experiments contributes to the robustness of the approach [64]. It is noteworthy that a method for adding 96 samples to a single glass slide is commercially available, however it remains necessary to image each comet one-by-one, and so the CometChip is approximately two orders of magnitude faster.

In conclusion, here we have demonstrated the efficacy of the CometChip for quantifying DNA damage and repair in many different tissues from mice exposed to two DNA methylating agents, MMS and NDMA. Furthermore, we applied the DNA glycosylases Fpg and hAAG to reveal the presence of unrepaired DNA bases. Results show that the CometChip can be used for the in vivo comet assay and that the platform is a powerful tool for increasing throughput, which is helpful for analyzing many tissues and multiple timepoints in parallel. As expected, tissues with higher expression of CYP2E1 showed significant levels of NDMA-induced DNA damage, whereas tissues with negligible CYP2E1 did not exhibit DNA damage following NDMA exposure. NDMA contamination is widespread and there are currently many sources of NDMA exposure to humans warranting the need to understand the DNA damaging consequences of exposure. The studies presented here provide insights into the DNA damaging effects of NDMA exposure in mice. Overall, these studies establish the in vivo CometChip as a novel approach for assessment of DNA damage in fresh or frozen tissues and opens doors to future studies of a wide range of DNA damaging agents that are relevant to medicine and public health. 

## 4. Materials and Methods

### 4.1. Chemicals and Reagents

TritonX-100 (cat. T8787), Tris base (cat. 93352), Tris-HCl (cat. T3253), HEPES (cat. H4034), bovine serum albumin (BSA) powder (cat. A3059), methyl methanesulfonate (MMS) (cat. 129925), dimethyl sulfonate (DMSO) (cat. D8418), potassium hydroxide (KOH) (484016), ethylenediaminetetraacetic acid (EDTA) (03620), ammonium sulfate ((NH_4_)_2_SO_4_), (A4418), magnesium sulfate (MgSO_4_), (M7506), potassium hydroxide (KOH) (cat. 484016) and Dulbecco’s phosphate-buffered saline (PBS) (cat. 14190144) were obtained from Millipore Sigma, St. Louis, MO. Normal melting point agarose (cat. 16500100), low melting point agarose (cat. 16520050), Hank’s Balanced Salt Solution (HBSS) (cat. 14025092), high-glucose Dulbecco’s modified Eagle’s medium (DMEM) (cat. 11965092), 200 mM L-glutamine (cat. 25030081), 10,000 units(U)/mL Pen-Strep (cat. 15140122), 0.25% Trypsin-EDTA with phenol red (cat. 25200) were purchased from Thermo Fisher Scientific, Waltham, MA. Fpg enzyme (cat. M0240) and 10,000 units/mL hAAG enzyme (cat. M0313) were purchased from New England Biolabs (NEB), Ipswich, MA. EDTA disodium salt dihydrate (Na_2_EDTA) (cat. MK772704) and potassium chloride (KCl) (cat. 6858) were purchased from VWR, Radnor, PA. Fetal bovine serum (FBS) was obtained from Atlanta Biologicals, Inc., Flowery Branch, GA, USA. 

### 4.2. Cell Culture

Both the mouse embryonic fibroblasts (MEF) and HepG2 cells were cultured in 37 °C incubator with 5% CO_2_. HepG2 and MEF cells were cultured in high DMEM supplemented with 10% FBS, 1X Pen-Strep and 1X L-glutamine. HepG2 cells (ATCC HB-8065), an immortalized cell line derived from human hepatocellular carcinoma, were obtained from American Type Culture Collection (Manassas, VA, USA). The WT MEFs used here were previously described [65].

### 4.3. MMS Treatment of MEFs and HepG2 Cells

Exponentially growing cells were plated in a tissue cultured 96-well plate at a density of 40,000 cells/well 24 h before treatment. MMS was diluted in DMSO, and cells were treated for 1 h in a 37 °C incubator with 5% CO_2_. Negative controls were treated with complete media with corresponding DMSO concentrations. After treatment, cells were trypsinized and loaded onto the CometChip. After cell loading, the alkaline comet assay was performed as described below.

### 4.4. Animals

All the experimental mice used were on a C57BL6 genetic background. The mice were maintained in AAALAC-certified animal care facilities and provided standard food and water. 

### 4.5. MMS Treatments of Mice

Four groups of mice (with *n* = 3 for each group) were treated with MMS via intraperitoneal (IP) injection at 15 days of age. The four cohorts included sham, 5 mg/kg, 25 mg/kg and 50 mg/kg. Three hours after sham or MMS treatment, mice were euthanized according to AVMA guidelines. Liver, lungs, kidneys, pancreas, spleen and brain were removed and processed as described below.

### 4.6. NDMA Treatments of Mice

NDMA was synthesized as previously described [66]. Mice were exposed to two separate injections, wherein 3.5 mg/kg NDMA in 10 μL saline was given at 8 days of age and 7 mg/kg in 20 μL saline was given at 15 days of age. Sham- and NDMA-treated mice were euthanized according to AVMA guidelines and tissues were harvested at 24 h, 48 h, 5 days and 10 weeks post-injection. Liver, lungs, kidneys, pancreas, and spleen were collected and processed as described below.

### 4.7. Tissue Sample Processing 

Organs collected at necropsy were immediately flash frozen in liquid nitrogen and stored at −80 °C until ready for processing. The MMS-exposed tissues were frozen for ~2 to 4 weeks before analysis, while the NDMA-exposed tissues were frozen for ~6 months to 2 years prior to analysis. To obtain cell suspensions from the frozen animal tissues, ~20 mg of each tissue was transferred from the −80 °C freezer into a 1.5 mL tube containing 1 mL cold tissue mincing solution (20 mM Na_2_EDTA and 10% DMSO in HBSS [Mg^2+^- and Ca^2+^-free]) at pH 7.5 (except for pancreas, which was suspended in 600 μL mincing solution). The tissues were gently minced using dissecting micro scissors and passed through a 40-μm filter, and the single-cell suspensions were kept on ice until loading onto the CometChip. The spleen tissue cell suspensions were further diluted (1:1 ratio) with tissue mincing solution before loading onto the chip. Subsequently, 50 μL of the cell suspensions were loaded onto the CometChip.

### 4.8. Alkaline Comet Assay Using the CometChip

Gels were either pre-made using a mold or purchased commercially from BioTechne. Cells were allowed to load into the microwells by gravity and excess cells were washed off with PBS by shear force. The chip containing the cells was then covered with a layer of 1% (*w*/*v*) LMP agarose in PBS and allowed to gelate at room temperature for 3 min, followed by 2 min at 4 °C (7 mL of LMP agarose was enough to cover a full CometChip). Cells were then encapsulated and immediately lysed via incubation in alkaline lysis buffer overnight at 4 °C (2.5 M NaCl, 100 mM Na_2_EDTA, 10 mM Trizma^®^ Base, and 1% (*v*/*v*) Triton X-100 at pH ~10). Following lysis, unwinding was performed via incubation in alkaline unwinding buffer (0.3 M NaOH and 1 mM Na_2_EDTA at pH ~13.5) for 40 min at 4 °C, and then the DNA was electrophoresed in the same buffer for 30 min at a constant voltage of 1 V/cm and ~300 mA. For enzyme-modified assay, the chip was incubated with enzyme buffer, to which either Fpg or hAAG was added, as described in the following section. Following electrophoresis, the CometChip was submerged and washed 3 times in neutralization buffer (0.4 M Trizma^®^ HCl at pH ~7.5). The DNA was then stained with 1X (0.01% (*v*/*v*) of 10,000X stock) SYBR Gold in PBS for 15 min at room temperature, protected from light using aluminum foil. Fluorescent images of the comets were captured at 4X magnification using a fluorescent microscope (Nikon Ti2-E inverted microscope). Image acquisition was performed by using the automatic scanning function, and the comet images were analyzed using the Trevigen Comet Analysis software. 

### 4.9. Fpg Enzyme Incubation

Chips were equilibrated in Fpg buffer (40 mM HEPES, 0.1 M KCl, 0.5 mM EDTA, 0.2 mg/mL BSA, pH 8.0) by washing 3X (5 min each) in the buffer. The chip was then either incubated in the buffer or with the Fpg enzyme (1:10,000 dilution in Fpg buffer) by adding 50 μL of Fpg buffer or Fpg buffer + Fpg enzyme into each CometChip macrowell. The chips were incubated for 1 h at 37 °C. To terminate the reaction, the chips were submerged in cold PBS and immediately transferred to cold alkaline unwinding buffer, after which the remaining steps of the comet assay were performed as described above.

### 4.10. hAAG Enzyme Incubation

Chips were equilibrated in hAAG buffer (20 mM Tris-HCl, 10 mM (NH_4_)_2_SO_4_, 10 mM HCl, 2 mM MgSO_4_, 0.1% Triton X-100, pH 8.8) by washing 3X (5 min each) in the buffer. The chip was then either incubated in the buffer or with the hAAG enzyme (10 U in hAAG buffer) by adding 50 μL of hAAG buffer or hAAG buffer + hAAG enzyme into each CometChip macrowell. The chips were incubated for 1 h at 37 °C. To terminate the reaction, the chips were submerged in cold PBS and immediately transferred to cold alkaline unwinding buffer and remaining steps of the comet assay performed as described above.

## Data Availability

The CometChip images are available upon request.

## Supplementary Materials

The supporting information can be downloaded at: https://www.mdpi.com/article/10.3390/ijms231911776/s1.
