# Supplementary material for: Novel In Vivo CometChip Reveals NDMA-Induced DNA Damage and Repair in Multiple Mouse Tissues
Source: Int J Mol Sci. 2022 Oct 4;23(19):11776. doi: 10.3390/ijms231911776 (PMC9569458; doi:10.3390/ijms231911776)
Supplement: Supplementary file 1 [file ijms-23-11776-s001.zip › ijms-1871877-supplementary.pdf]

International Journal of *Molecular Sciences*

Supplementary materials

Novel *in vivo* CometChip Reveals NDMA-induced DNA Damage and Repair in Multiple Mouse Tissues

Norah A. Owiti[1], Joshua J. Corrigan[1], Lee J. Pribyl[1], Jennifer E. Kay[2] and Bevin P. Engelward[1]

[1] Department of Biological Engineering, Massachusetts Institute of Technology, Cambridge, MA 02139
[2] Silent Spring Institute, Newton, MA 02460

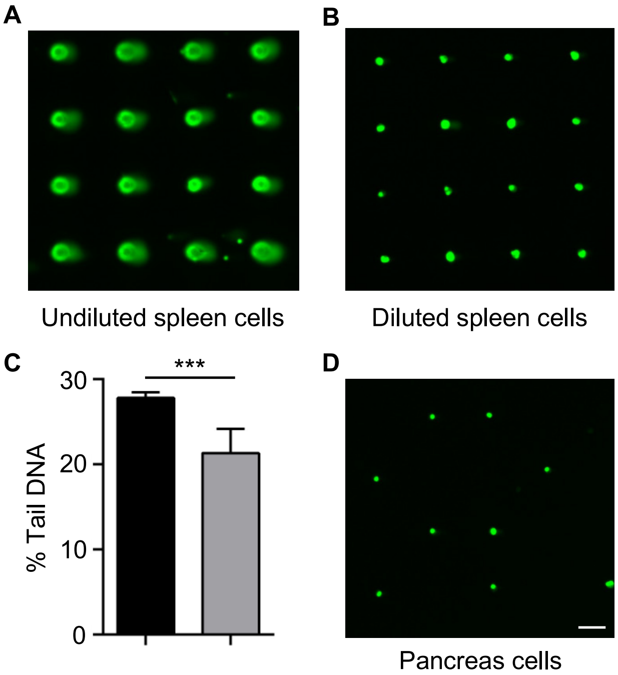

Supplementary Figure S1. Representative images of spleen and pancreas cells loaded onto the CometChip. Single cell suspensions from the spleen and pancreas of control mice were loaded onto the CometChip and analyzed with the alkaline comet assay. **(A)** (*Left*) Representative image of cells from the spleen after the comet assay was performed with undiluted cells (use of 1 mL of mincing solution). (*Right)* Representative image of cells from the spleen after the comet assay was performed with diluted cells. (**B**) Bar graph showing quantification of (A). (**C**) Representative image of cells from the pancreas loaded onto the chip. Scale bar = 100 µm.

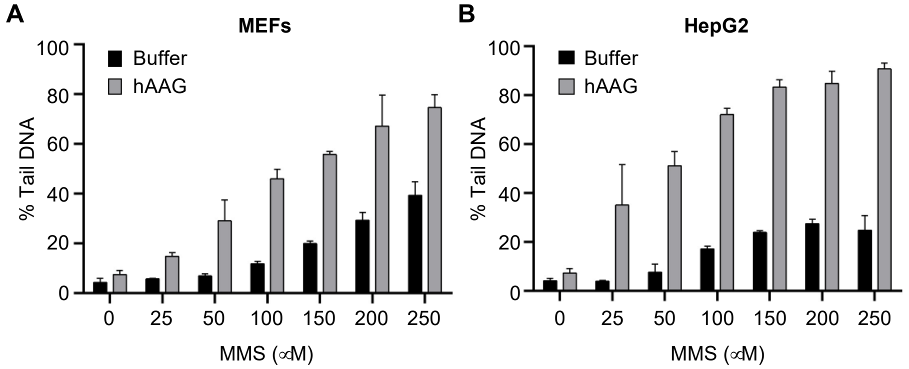

Supplementary Figure S2. hAAG enzyme reveals MMS-induced base lesions in MEFs and HepG2 cells. MEFs and HepG2 cells were treated with various concentrations of MMS for 1 hour and DNA damage was analyzed with the alkaline CometChip modified with hAAG enzyme or hAAG buffer. **(A)** Results with MEFs. (**B**) Results with HepG2 cells. Percent tail DNA represented as mean of three replicates ± SD.

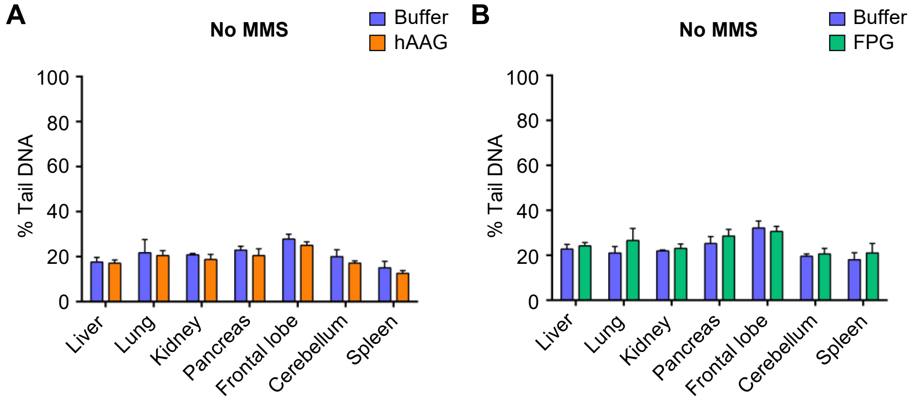

Supplementary Figure S3. Control tissues do not show an increase in DNA damage following incubation with hAAG or Fpg. Tissues from sham-treated (DMSO) mice were analyzed with enzyme-modified alkaline CometChip. Results represent DNA damage quantified in various tissues modified with **(A)** hAAG enzyme or hAAG buffer and (**B**) Fpg enzyme or Fpg buffer. Percent tail DNA represented as mean of three mice ± SD.

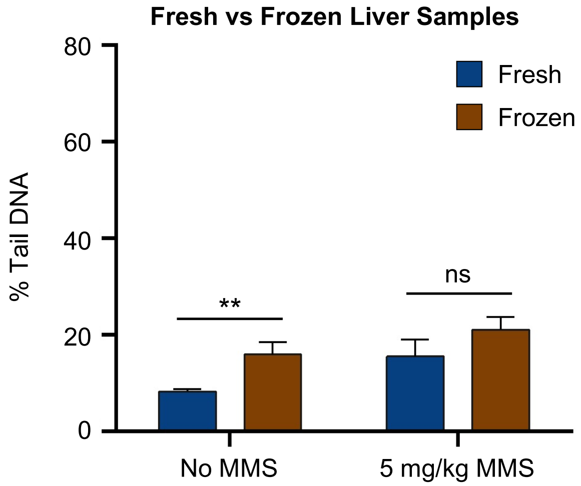

Supplementary Figure S4. Freezing tissues increases background DNA damage levels. Fresh and frozen liver tissues from sham-treated or MMS-treated mice (exposed to 5 mg/kg of MMS) were analyzed using the alkaline CometChip for strand breaks. Percent tail DNA represented as mean of three mice ± SD. Unpaired Student's *t*-test, $^{**}p < 0.01$.

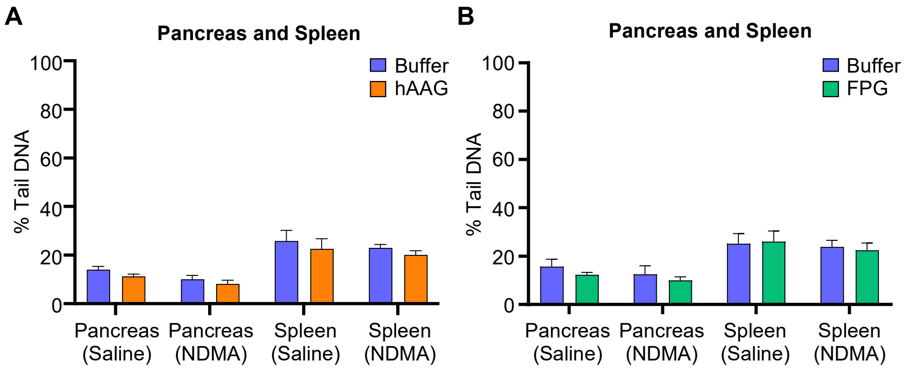

Supplementary Figure S5. Use of hAAG and Fpg enzymes to reveal modified bases in the pancreas and spleen following NDMA exposure. Pancreas and spleen from sham- or NDMA-treated mice (exposed to 10 mg/kg of NDMA) were collected 24 hours after NDMA exposure. The tissues were analyzed with enzyme-modified alkaline CometChip. Results show DNA damage in the pancreas and spleen following modifications with **(A)** hAAG enzyme or hAAG buffer and **(B)** Fpg enzyme or Fpg buffer. Percent tail DNA represented as mean of three mice ± SD.
